# The Importance of Positive Events When Living With Chronic Pain

**DOI:** 10.1093/geroni/igaa057.990

**Published:** 2020-12-16

**Authors:** Julie Kircher, Susan Charles, Nancy Sin, David Almeida

**Affiliations:** 1 University of California, Irvine, Irvine, California, United States; 2 BSS, Irvine, California, United States; 3 University of British Columbia, Vancouver, British Columbia, Canada; 4 Pennsylvania State University, University Park, Pennsylvania, United States

## Abstract

Chronic pain is a common condition in later life that is related to high levels of anxiety and depression. One reason why chronic pain is related to affective distress is that this condition may prevent people from deriving the same positive emotions from enjoyable activities. Few studies, however, have examined how exposure and reactivity to daily events differ by chronic pain status. We hypothesized that those with chronic pain will have less exposure and less positive affect reactivity to positive daily events compared to those without chronic pain. Participants from the diary substudy of MIDUS (N = 1,733; nChronicPain = 658, nNoPain = 1,075; M = 56 years-old) completed eight interview days. Chronic pain status was unrelated to the frequency of positive events. Multi-level models revealed that although people with chronic pain had lower levels of daily positive affect, they reacted more positively to daily events (γ = -.033, SE = .010, p < .0001). As a result, levels of daily positive affect on days when people experienced a positive event did not vary by pain status (MChronicPain = 2.73, MNoPain = 2.75). People with chronic pain averaged higher levels of daily negative affect compared to people without chronic pain (MChronicPain = .21, M NoPain =.20), but, on days when they experience a positive event, those with chronic pain had a greater decrease in their negative affect. Findings suggest that positive events impact those with chronic pain more than they do individuals without chronic pain.

